# Acute Abdomen with Periumbilical Erythema

**DOI:** 10.5334/jbsr.1566

**Published:** 2018-07-05

**Authors:** Petiot Alice, Vassiliki Pasoglou

**Affiliations:** 1 Cliniques Universitaires Saint Luc, BE

**Keywords:** Acute abdomen, Malone, Appendicitis, CT scan

## Abstract

A 33-year-old man with a history of a Malone Antegrade Continence Enema Procedure presented to the Emergency Department with right lower abdominal pain. Computed Tomography (CT) of the abdomen revealed an appendicitis of the appendicostomy with an associated appendicolith.

A 33-year-old man presented to the emergency department with right-lower abdominal pain, high-grade fever (40°C), nausea, and chills. The skin around his umbilicus was erythematous (Figure 1) and itchy. The physical examination revealed a rebound tenderness, guarding and rigidity upon palpation of the right iliac fossa and periumbilical region. The patient had a history of a Malone Antegrade Continence Enema Procedure (MACE), for intractable fecal incontinence. The latest appendicostomy catheterization was ten years ago, followed by spontaneous closure.

**Figure 1:**
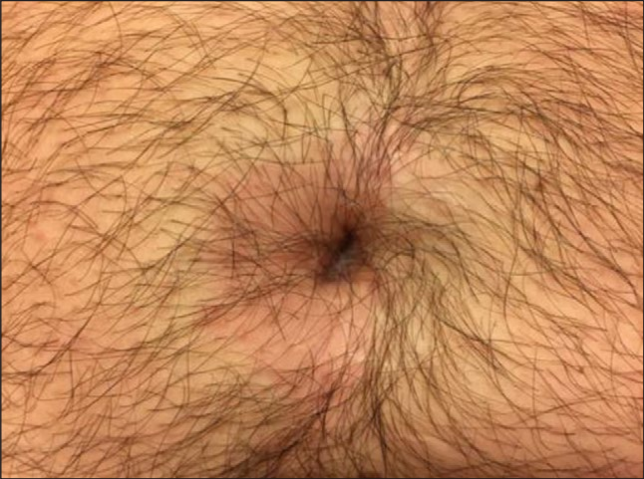
Periumbilical erythema.

Computed tomography (CT) of the abdomen revealed a dilated and thickened intestinal structure, extending from the caecum toward the umbilicus and containing a calcified deposit (Figure 2). These findings were consistent with appendicitis of the appendicostomy with appendicolith.

**Figure 2:**
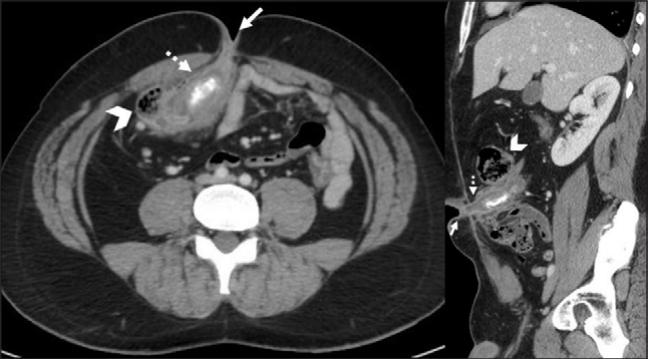
Coronal reformatted and axial contrast medium-enhanced CT-scan images showing dilated and thickened intestinal structure (dotted arrow) between the caecum (arrowhead) and the umbilicus (arrow), containing a calcified deposit.

During MACE, the appendix is surgically connected to the umbilicus to allow administration of enemas to the right colon. This procedure is performed in patients with fecal incontinence or chronic constipation when conventional treatments such as dietary modifications, oral laxatives, and rectal enemas have been ineffective. This surgical procedure generally has an excellent outcome with marked improvement to quality of life. However, complications include stomal site infection, leakage, mucosal prolapse, and stenosis as well as troubles with stomal catheterization [1].

Our patient underwent successful conservative treatment with antibiotics and was scheduled for surgery in three months.
